# Supplementary material for: The C. elegans homolog of human panic-disorder risk gene TMEM132D orchestrates neuronal morphogenesis through the WAVE-regulatory complex
Source: Mol Brain. 2021 Mar 16;14:54. doi: 10.1186/s13041-021-00767-w (PMC7962252; doi:10.1186/s13041-021-00767-w)
Supplement: Supplementary file 1 — Additional file 1: Fig. S1. C. elegans TMEM-132 is a member of the evolutionarily conserved TMEM132 protein family. Fig. S2. C. elegans TMEM-132 maintains morphologically complex PVD, ADE but not AWC neurons. Fig. S3 Efficacy of RNAi in neurons by feeding from bacteria. Fig. S4. Enrichment of mammalian TMEM132D expression in the brain and claustral neurons. Fig. S5. TMEM132D regulates F-actin abundance. [file 13041_2021_767_MOESM1_ESM.pdf]

Fig. S1

a

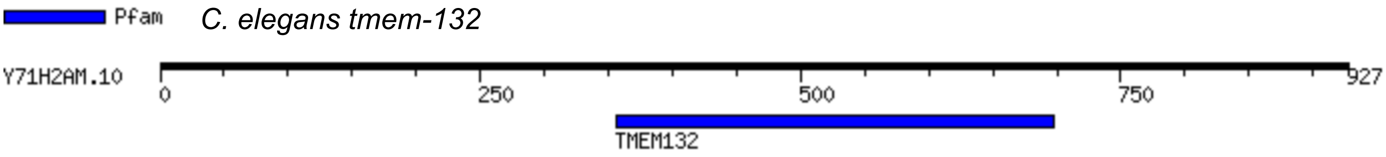

Pfam (1 motif)

Pfam	Position(Independent E-value)	Description
TMEM132	356..698(2e-85) Detail	PF16070, Transmembrane protein family 132

b

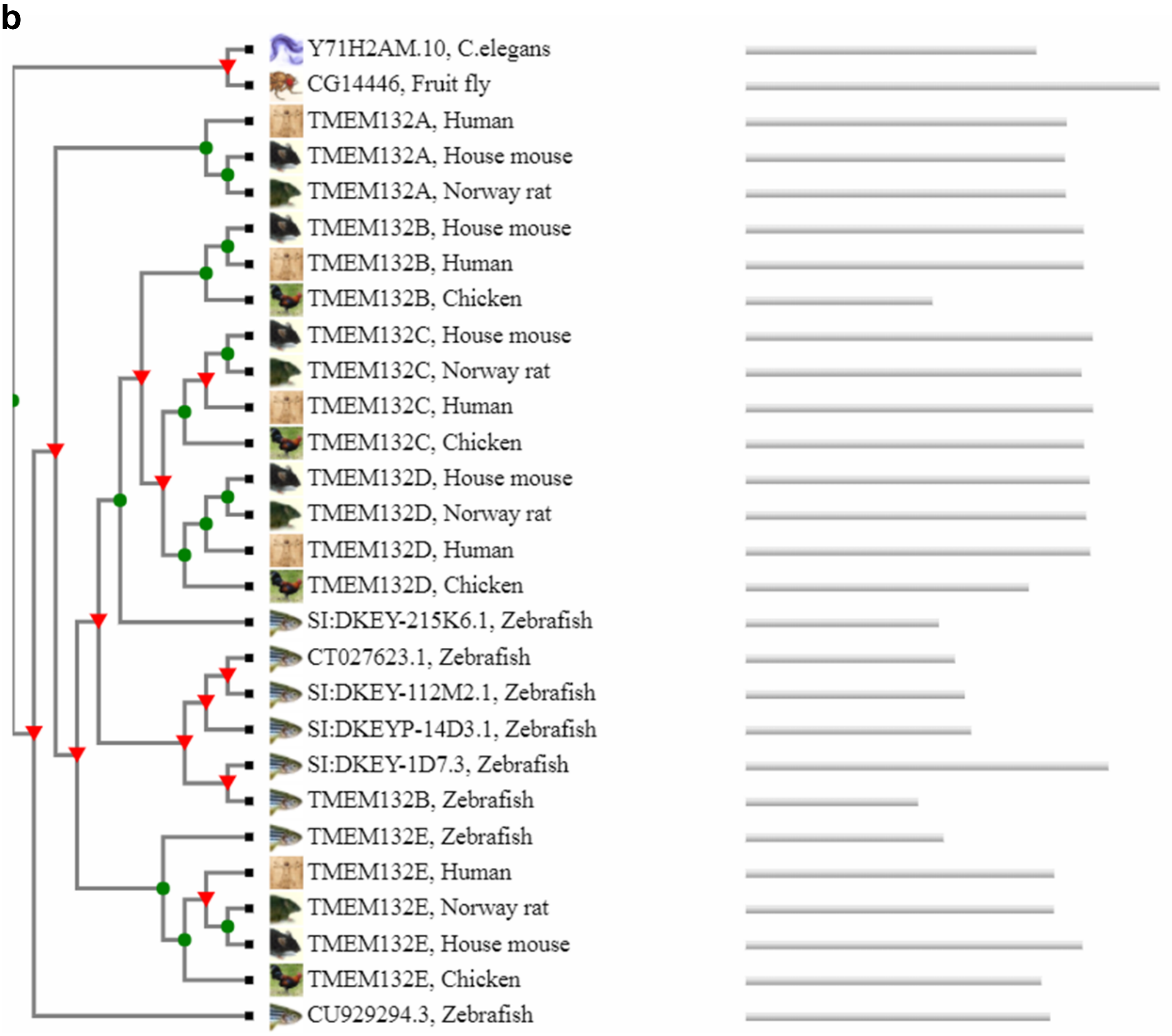

Fig. S2

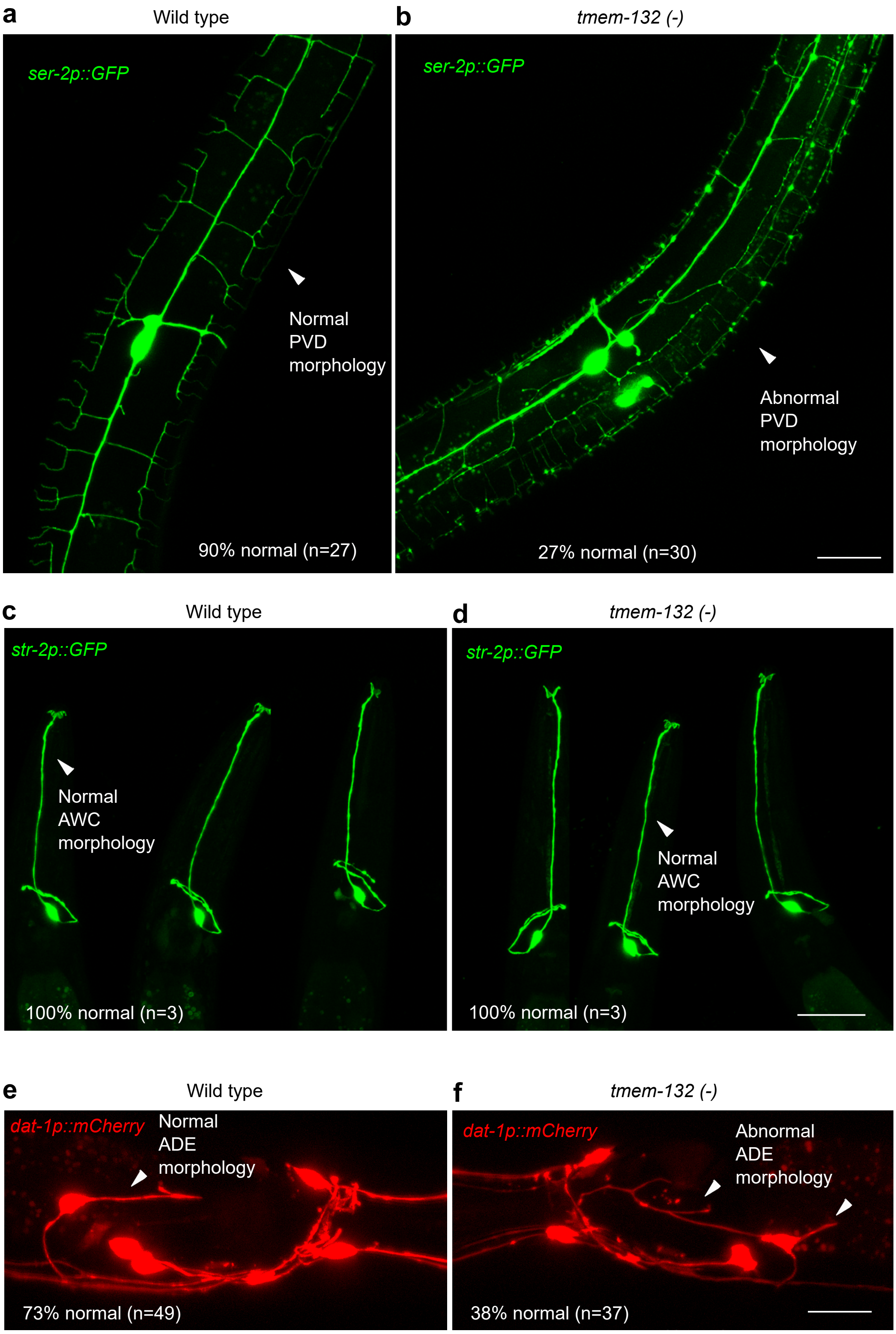
a
Wild type
ser-2p::GFP
Normal
PVD
morphology
90% normal (n=27)
b
tmem-132 (-)
ser-2p::GFP
Abnormal
PVD
morphology
27% normal (n=30)
c
Wild type
str-2p::GFP
Normal
AWC
morphology
100% normal (n=3)
d
tmem-132 (-)
str-2p::GFP
Normal
AWC
morphology
100% normal (n=3)
e
Wild type
dat-1p::mCherry
Normal
ADE
morphology
73% normal (n=49)
f
tmem-132 (-)
dat-1p::mCherry
Abnormal
ADE
morphology
38% normal (n=37)

Fig. S3

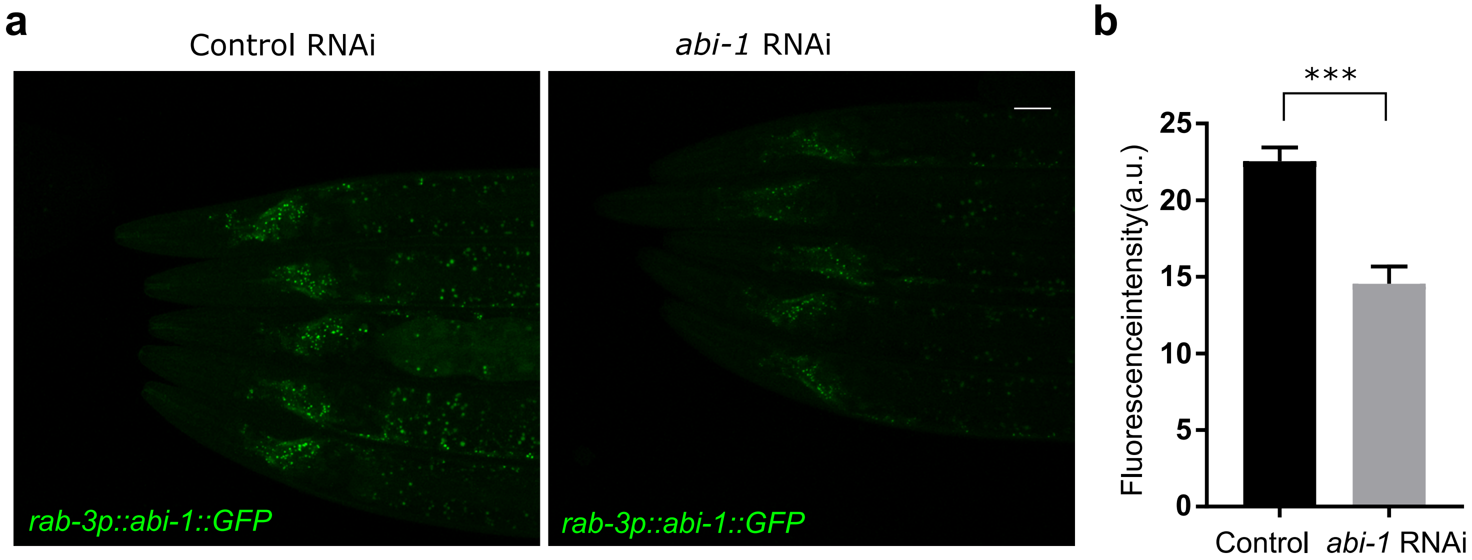
a
Control RNAi
abi-1 RNAi
rab-3p::abi-1::GFP
rab-3p::abi-1::GFP
b
***
Fluorescenceintensity(a.u.)
25
20
15
10
5
0
Control
abi-1 RNAi

Fig. S4

a

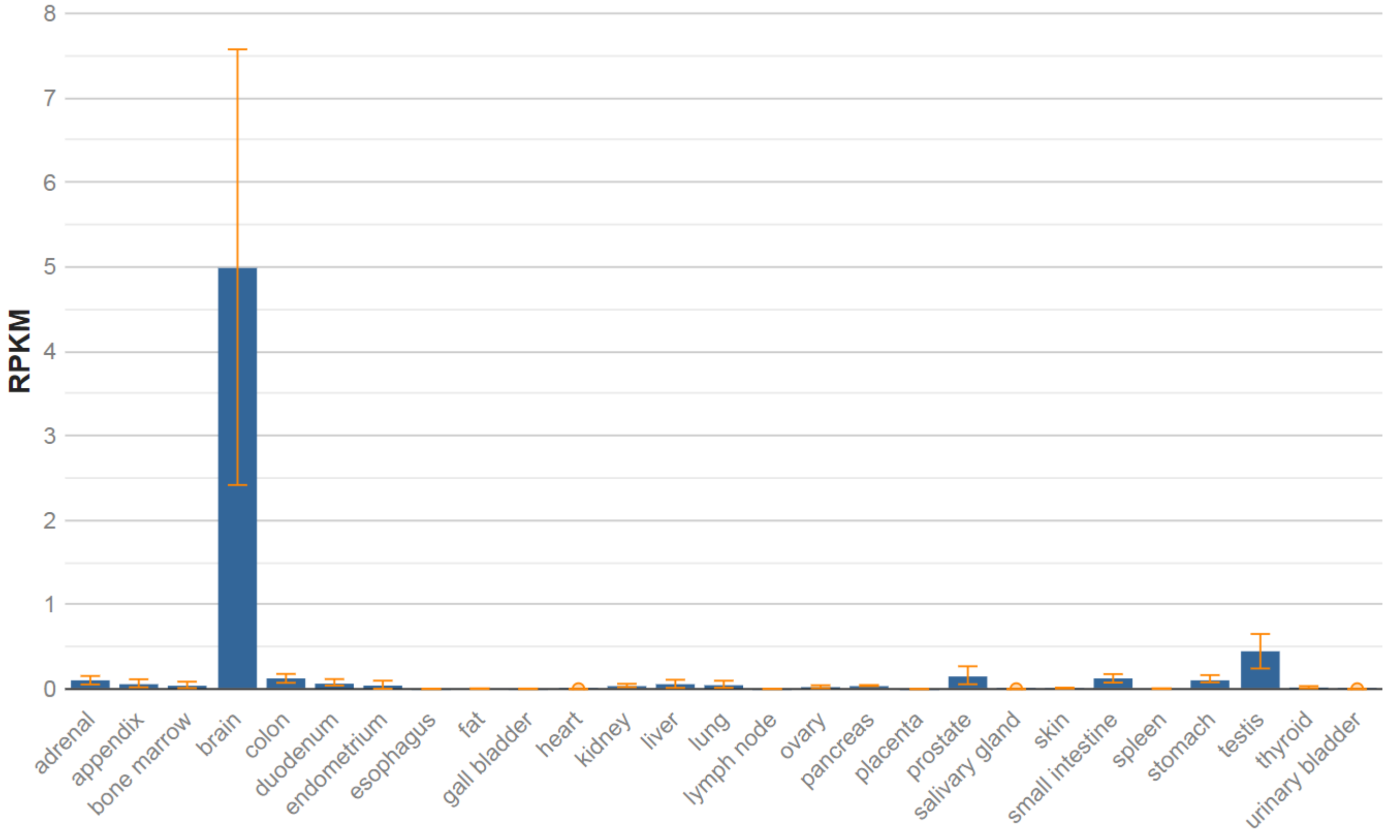

RPKM
8
7
6
5
4
3
2
1
0
adrenal
appendix
bone marrow
brain
colon
duodenum
endometrium
esophagus
fat
gall bladder
heart
kidney
liver
lung
lymph node
ovary
pancreas
placenta
prostate
salivary gland
skin
small intestine
spleen
stomach
testis
thyroid
urinary bladder

b

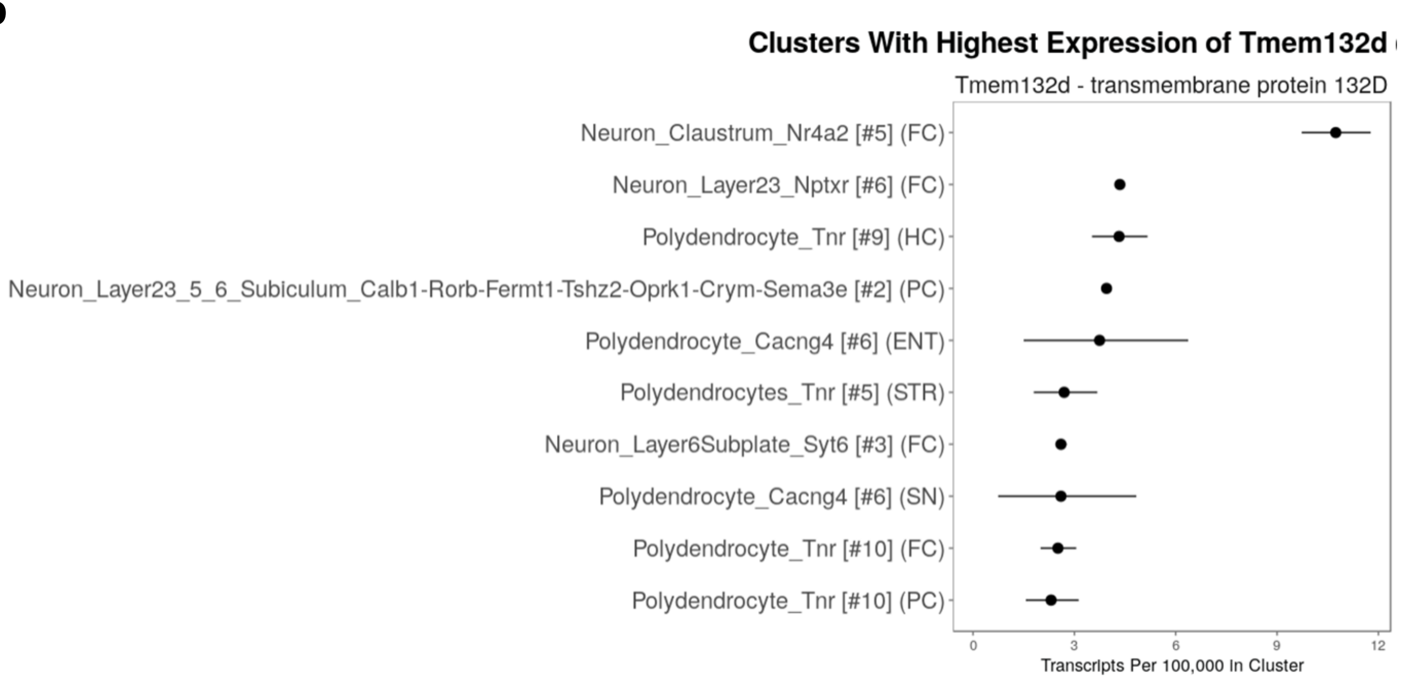

Clusters With Highest Expression of Tmem132d
Tmem132d - transmembrane protein 132D
Neuron_Claustrum_Nr4a2 [#5] (FC)
Neuron_Layer23_Nptxr [#6] (FC)
Polydendrocyte_Tnr [#9] (HC)
Neuron_Layer23_5_6_Subiculum_Calb1-Rorb-Fermt1-Tshz2-Oprk1-Crym-Sema3e [#2] (PC)
Polydendrocyte_Cacng4 [#6] (ENT)
Polydendrocytes_Tnr [#5] (STR)
Neuron_Layer6Subplate_Syt6 [#3] (FC)
Polydendrocyte_Cacng4 [#6] (SN)
Polydendrocyte_Tnr [#10] (FC)
Polydendrocyte_Tnr [#10] (PC)
0
3
6
9
12
Transcripts Per 100,000 in Cluster

Fig. S5

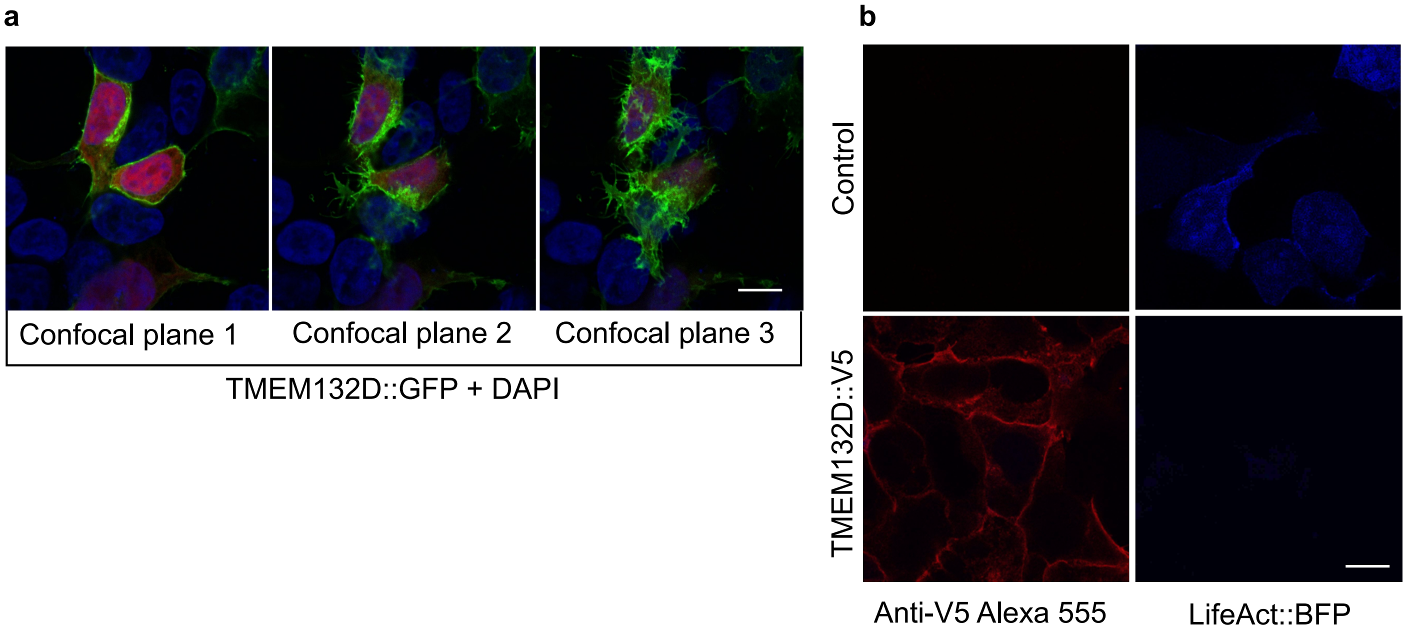
a
Confocal plane 1
Confocal plane 2
Confocal plane 3
TMEM132D::GFP + DAPI
b
Control
TMEM132D::V5
Anti-V5 Alexa 555
LifeAct::BFP
