# Supplementary material for: The C. elegans homolog of human panic-disorder risk gene TMEM132D orchestrates neuronal morphogenesis through the WAVE-regulatory complex
Source: Mol Brain. 2021 Mar 16;14:54. doi: 10.1186/s13041-021-00767-w (PMC7962252; doi:10.1186/s13041-021-00767-w)
Supplement: Supplementary file 2 — Additional file 2: Table S1. List of genes encoding putative TMEM132D interactors identified from yeast-two-hybrid screens. [file 13041_2021_767_MOESM2_ESM.pdf]

Supplementary Table 1. List of genes encoding putative TMEM132D interactors identified from yeast-two-hybrid screens.

Gene Name	Occurrence frequency	Gene Name	Occurrence frequency
NCKAP1	3	*HDAC2*	1
RNF2	1	*TNFAIP8*	1
HDAC2	1	*SDHB*	1
NDUFS5	1	*PCLO*	2
PLSCR2	2	*PNISR*	2
STK39	1	*ATP7A*	1
HOXA9	1	*FAM35A*	1
TMED7-TICAM2	2	*PTPN2*	1
PTPRD	1	*RAB6A*	3
CD59	2	*ANKRD31*	1
NM_005746.2	1	*PGM1*	1
ANKRD36	2	*SEC22B*	1
PNISR	2	*TUBGCP5*	3
CMPK1	1	*HEBP2*	2
UMOD	1	*SLC35E2*	1
TPST1	1	*HNRNPUL1*	1
TMED5	2	*ZNF302*	2
SRSF5	1	*TOB2*	1
IDI2-AS1	1	*COMMD1*	2
ENOPH1	6	*LNX1*	1
H3K27Ac	1	*TRAPPC8*	1
EFEMP1	2	*TCF12*	1
VPS13C	1	*PAIP1*	1
BNIP3L	1	*CNGB3*	1
PRPF40A	9	*GFM2*	1
COX11	1	*MIR181A1HG*	1
USP32P2	2	*NAMPT*	1
FMO2	1	*MME*	1
RNF2	1	*ZBTB20*	2
SEP_15	2	*RCBTB2*	1
USP32P2	3	*ATP1B1*	1
CNTN1	2	*SVIL*	1
TRAPPC11	3	*CTD-2303H24.2*	1
CTD-2175A23.1	2	*UBA5*	1
IL13RA1	1	*CADPS2*	1
PCCA	2	*CPD*	1
NPAS3	1	*CCDC85A*	1
PRMT3	1	*SAMD12*	1
